# Research progress in ultrasound use for the diagnosis and treatment of cerebrovascular diseases

**DOI:** 10.6061/clinics/2019/e715

**Published:** 2019-02-27

**Authors:** Li Yan, Xiaodong Zhou, Yu Zheng, Wen Luo, Junle Yang, Yin Zhou, Yang He

**Affiliations:** I Department of Ultrasonography, Xijing Hospital, The Fourth Military Medical University, Xi’an , China.; II Department of Ultrasonography, Xi’an Central Hospital, The Third Affiliated Hospital of JiaoTong University, Xi’an, China.; III Department of CT & MRI, Xi’an Central Hospital, The Third Affiliated Hospital of JiaoTong University, Xi’an, China.; IV Department of General Surgery, Xi'an Medical University, Xi'an, China.

**Keywords:** Ultrasound, Vascular Imaging, Cerebrovascular Event, Carotid Artery, Ultrasound Contrast, Thrombolysis

## Abstract

Cerebrovascular diseases pose a serious threat to human survival and quality of life and represent a major cause of human death and disability. Recently, the incidence of cerebrovascular diseases has increased yearly. Rapid and accurate diagnosis and evaluation of cerebrovascular diseases are of great importance to reduce the incidence, morbidity and mortality of cerebrovascular diseases. With the rapid development of medical ultrasound, the clinical relationship between ultrasound imaging technology and the diagnosis and treatment of cerebrovascular diseases has become increasingly close. Ultrasound techniques such as transcranial acoustic angiography, doppler energy imaging, three-dimensional craniocerebral imaging and ultrasound thrombolysis are novel and valuable techniques in the study of cerebrovascular diseases. In this review, we introduce some of the new ultrasound techniques from both published studies and ongoing trials that have been confirmed to be convenient and effective methods. However, additional evidence from future studies will be required before some of these techniques can be widely applied or recommended as alternatives.

## ULTRASOUND IN CAROTID ATHEROSCLEROSIS

As our understanding of the pathogenesis of carotid atherosclerosis and stroke increases, the importance and necessity of carotid ultrasonography have become increasingly apparent in clinical practice. Carotid ultrasonography allows for the real-time dynamic monitoring of the vascular lumen and tunica intima, visual display of the hemodynamic changes in the carotid artery and accurate measurement of the degree of carotid artery stenosis ( 1 , 2 ). In addition, carotid ultrasonography can be used to determine the location, size and nature of plaques, assess the stability of plaques and evaluate the efficacy of drugs during regular follow-up examinations ( 3 - 5 ).

Ultrasound techniques employed to examine and evaluate carotid artery lesions include the conventional B-mode ultrasound, color Doppler ultrasound, spectral Doppler ultrasound, vascular enhancement technology (VET), carotid plaque contrast agent enhancement, intravascular ultrasound (IVUS) and echo-tracking techniques.

**Figure 1:**
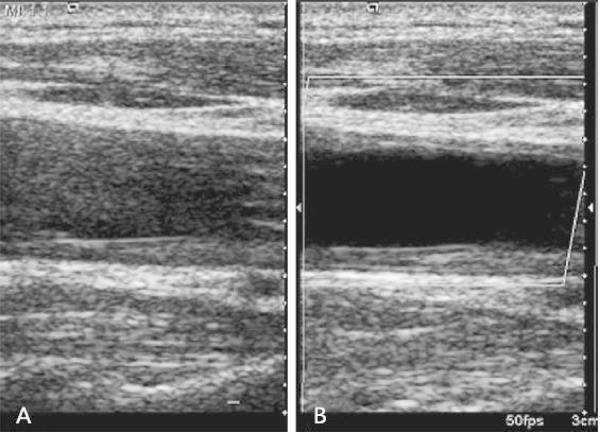
- Grayscale 2D ultrasound shows an obscured anterior wall and sound artifacts in the lumen (A), while a VET image shows a clear lumen and a thickened endo-medial tunica of the anterior and posterior wall (B).

**Figure 2:**
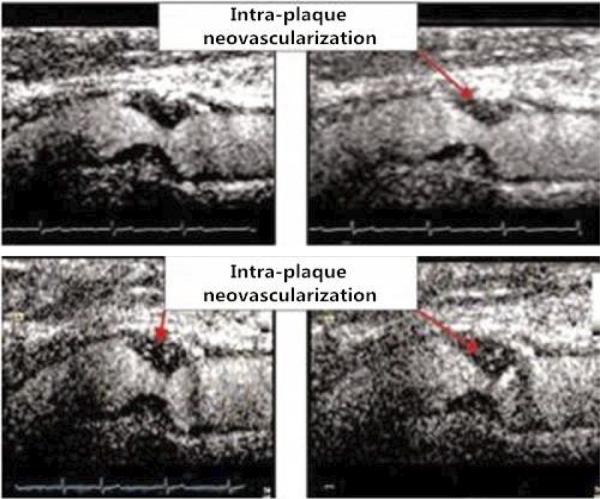
- A sequence of images obtained following the intravenous injection of ultrasound contrast indicates the presence of intra-plaque neovascularization in the sequential frames (Grade 2.5, extensive neovascularization). The presence and degree of neovascularization were determined by surgery and laboratory testing.

In clinical practice, high-frequency ultrasound is highly useful for examination of the carotid artery. Conventional two-dimensional ( 2 D) Doppler, color Doppler and spectral Doppler ultrasound imaging are the preferred examination methods in both morphological and functional studies ( 6 ). In morphological studies, ultrasound has been employed to image the tunica intima and tunica media of blood vessels and diagnose plaques, thrombosis and other vascular diseases ( 7 , 8 ). In functional studies, ultrasound has been used to examine vascular endothelial function ( 9 ). Due to the increasing understanding of disease theories, clinical techniques are being updated and improved synchronously. A comparative study conducted by Liu et al. ( 6 ) demonstrated that the application of VET improves the ability and sensitivity of ultrasound to assess the microvasculature and produces a clearer view of the vessel lumen and wall structure ( Figure 1 ). In recent years, a fundamental cause of many unpredictable arterial incidents and serious clinical consequences has been the inability to accurately evaluate the risk of atherosclerotic plaques in clinical practice ( 10 - 12 ). Therefore, the ability to conduct multi-angle and multi-level analyses of plaques using a combination of novel ultrasonic evaluation parameters and contrast-enhanced ultrasound (CEUS) imaging of atherosclerotic plaque neovascularization will provide richer diagnostic information for the evaluation of plaque stability ( 13 , 14 ). McCarthy et al. argued that plaque neovascularization is a clear sign of plaque instability and vulnerability ( 15 ). In addition, Nowik M et al. demonstrated that the enhanced angiographic signal at the periphery and interior of the plaque is related to plaque neovascularization and reflects the presence and extent of the local inflammatory response in the plaque ( 16 ). Furthermore, Shah et al. ( 17 ) determined that the enhanced angiographic signal of carotid plaque neovasculature is correlated with the extent of neovascularization, which was confirmed in surgical specimens ( 17 ) ( Figure 2 ).

With the continuous development of ultrasound imaging technology and the increasing accumulation of clinical diagnostic experience ( 18 ), carotid ultrasonography has become the most widely used method of detecting atherosclerosis in clinical practice ( 19 - 21 ). CEUS of the carotids is a further generalized method that is used to examine and evaluate the microvasculature of atherosclerotic plaques. Thus, this technique provides visual evidence for early clinical intervention and treatment of cerebrovascular diseases ( 22 , 23 ).

## ULTRASOUND IN CEREBRAL ARTERIAL OCCLUSION

### Transcranial Doppler (TCD) ultrasonography

Rapid and accurate diagnosis and evaluation of cerebrovascular diseases are important to reduce the incidence, morbidity and mortality of these conditions ( 24 , 25 ). TCD examination is a classic means of diagnosing cerebral blood vessels. TCD is a non-invasive method developed in the early 1980s that examines intracranial hemodynamics ( 26 ). In 1982, Aaslid et al. utilized this non-invasive technique for the first time to detect low-speed blood flow in basal cerebral arteries ( 27 ). Subsequently, TCD began to be applied clinically, and in recent years, TCD has developed rapidly.

TCD has been widely used in clinical practice. Due to its advantages, such as its non-invasiveness and facile operation, TCD has become increasingly important in the clinical diagnosis of ischemic cerebrovascular diseases in recent years ( 28 - 30 ). However, TCD is incapable of intuitively reflecting the structural morphology of cerebral vessels. It only indirectly reflects the state of the vasculature via examination of the blood flow velocity at the sampling points; however, the velocity of the blood flow itself is affected by many physiological factors ( 31 ). Moreover, due to the inadequate acoustic window, TCD has certain limitations in the diagnosis of cerebrovascular diseases.

Color and power Doppler TCD were developed based on TCD. Although they exhibit certain improvements and enhancements over TCD, the phenomenon of “high energy overflow”, which is attributed to the thinness of the intracranial vasculature and conventional angle-dependence of ultrasound examinations, still greatly influences the diagnosis of cerebrovascular diseases ( 32 - 34 ).

### Transcranial ultrasound angiography (tUSA)

**Figure 3:**
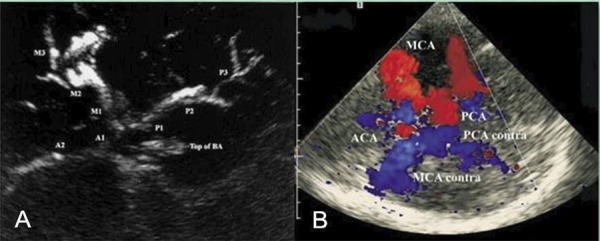
A. tUSA image of the circle of Willis and its branches after injection of ultrasound contrast agents. M1, M2, M3=middle cerebral artery segments; A1, A2=anterior cerebral artery segments; P1, P2, P3, P4=posterior cerebral artery segments; BS=brainstem; Top of BA=hyperechogenic distal part of the basilar artery. B. Contrast-enhanced transcranial ultrasound of the circle of Willis (axial scanning plane via the temporal bone window). Vessel delineation is diminished because of the strong contrast signal (“blooming”) after IV bolus injection of UCA. MCA=middle cerebral artery; MCA contra=middle cerebral artery contralateral; PCA=posterior cerebral artery; PCA contra=posterior cerebral artery contralateral; ACA=anterior cerebral artery.

Holscher et al. explored a novel ultrasound technique called tUSA ( 35 ), which uses contrast agents to generate clear and intuitive images of intracranial blood vessels similar to those obtained by conventional angiography ( 35 ) ( Figure 3 ). In addition, tUSA adopts low-frequency (1.0-2.0 MHz) and high-mechanical-index (1.1-2.0) ultrasound to obtain ideal image signals and utilizes a harmonic imaging technique to achieve satisfactory tissue and contrast imaging. Compared to digital subtraction angiography (DSA), tUSA possess several advantages. tUSA is easy to operate, virtually non-invasive and inexpensive. In addition, tUSA reflects the vascular condition dynamically and in real-time and is suitable for repeated examinations. Under emergency conditions, tUSA can be performed at the bedside. Wei et al. performed bilateral tUSA and DSA on 60 patients with clinically suspected cerebral arterial occlusion. Both tUSA and DSA were able to image the cerebral arteries and cerebral arterial circles in all patients. Twenty-eight patients were diagnosed with cerebral arterial occlusion using tUSA. Among them, 22 patients were diagnosed with middle cerebral artery (MCA) occlusion, and 6 patients were diagnosed with anterior cerebral artery (ACA) occlusion. However, only 26 patients were diagnosed with cerebral arterial occlusion using DSA. Among them, 22 patients were diagnosed with MCA occlusion, and 4 patients were diagnosed with ACA occlusion. Therefore, tUSA displayed a higher sensitivity and specificity in the diagnosis of cerebral arterial occlusion.

### Three-dimensional transcranial color-coded sonography (3D-TCCS) and contrast-enhanced3D transcranial color-coded sonography (3D-ceTCCS)

**Figure 4:**
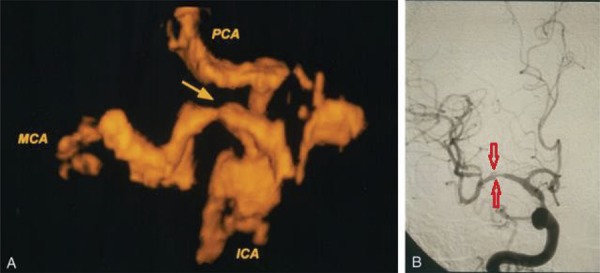
Anteroposterior images of an intracranial stenosis. A. A 3D power doppler image shows the circle of Willis with severe stenosis (arrow) in the MCA. ICA=internal carotid artery; MCA=middle cerebral artery; PCA=posterior cerebral artery. B. An angiogram confirmed severe stenosis (arrows) in the MCA stem.

Stroke is closely related to intracranial arterial stenosis and occlusion ( 36 ). 3D ultrasound utilizes reconstruction technology to more closely reflect the actual anatomical structure of the target area and allows for multi-angle intuitive examination and evaluation of the target area. Moreover, the objective 3D data generated by 3D ultrasound reduce the cognitive bias of the examiners and improve the accuracy of the examination. Klotzsch et al. ( 37 ) evaluated 36 stenotic or occlusive lesions in 26 patients with intracranial arterial stenosis or occlusion using 3D-ceTCCS and compared the results to those generated by DSA ( 37 ) ( Figure 4 ). Upon estimating the degree of stenosis, 3D-ceTCCS and DSA yielded the same diagnosis in 33 of 36 lesions. However, DSA indicated a diagnosis of partial occlusion when evaluating 3 lesions, whereas 3D-ceTCCS indicated a diagnosis of complete occlusion. Nevertheless, 3D-ceTCCS and DSA generated highly consistent diagnoses (k=0.86).

## ULTRASOUND IN THROMBOLYSIS

With the rapid development of medical ultrasound, ultrasound imaging technology has become increasingly incorporated in clinical practice for the treatment of cerebrovascular diseases ( 38 - 40 ). Thrombolytic therapy reduces the mortality of acute ischemic stroke, improves clinical outcomes of patients and reduces post-stroke disability by dissolving arterial thrombi ( 41 , 42 ). Ultrasound thrombolysis takes advantage of the effects of ultrasound itself and the thermal, mechanical and physicochemical effects produced by the sound waves ( 43 - 45 ). It causes a series of biological effects in the tissues to achieve the desired therapeutic effects ( 46 , 47 ). In 1976, Trubestein et al. demonstrated for the first time that ultrasound could destroy and immediately remove thrombi in blood vessels without causing apparent side effects or complications in animals ( 48 ).

Ultrasound thrombolysis includes IVUS thrombolysis and ultrasound-assisted extracorporeal thrombolysis. In recent years, researchers have conducted many in vivo experiments, in vitro experiments and clinical trials on ultrasound thrombolysis ( 49 , 50 ). The results show that noninvasive ultrasound within the clinically applicable intensity range significantly promotes the fibrinolytic effect of enzymes on fibrin and on the entire blood clot ( 51 ). Ultrasound promotes thrombolysis through the stimulation of enzymatic degradation instead of through the mechanical destruction of fibrin. Eggers et al. conducted a study on the application of ultrasound thrombolysis to treat acute ischemic stroke, which is a contraindication for recombinant tissue plasminogen activator (rt-PA) therapy ( 52 ). In the study, 15 subjects were randomly divided into a treatment group and a control group. The treatment group underwent ultrasound thrombolysis for over 1 hour. Four days later, the outcomes were evaluated. The rates of vascular recanalization and neurological improvement were significantly higher in the treatment group than in the control group. The study demonstrated that ultrasound thrombolysis is an effective treatment and provides a new treatment option for stroke patients who are not eligible for rt-PA-based thrombolytic therapy.

Despite the obvious advantages of ultrasound thrombolysis, the data available on its safety are limited ( 53 ). Further studies are needed to demonstrate the increased efficacy and safety of ultrasound thrombolysis compared to currently approved therapeutic options ( 54 ). Nevertheless, ultrasound has been attracting increasing attention as a novel thrombolytic regimen.

CEUS is a non-invasive, fast, efficient, reproducible and apparently very sensitive imaging modality ( 55 - 57 ). CEUS examination of the carotid artery is a new tool to improve the delineation of the vessel wall and to analyze atherosclerotic carotid lesions by detecting the presence and extent of plaque neovascularization and providing information on plaque vulnerability in patients at risk for developing symptomatic atherosclerosis ( 58 ). Despite its advantages, this technique has not been widely used and is highly dependent on operator experience ( 59 ). In addition, quantitative research is still needed.

Currently, ultrasound technology is developing rapidly and is becoming involved in an increasing number of fields. The application of ultrasound has expanded from traditional diagnosis to treatment and has achieved remarkable results. As the number of patients with brain diseases has increased, cranial sonography techniques have advanced from simple morphological imaging to complex functional recovery and treatment. We believe that ultrasound technology has great potential and will be a promising method for the diagnosis and treatment of cerebrovascular diseases in the future.
